# Effect of Tell-Show-Do Versus Audiovisual Distraction on Pediatric Dental Anxiety and Cooperation: A Prospective Non-randomized Study in Greece

**DOI:** 10.7759/cureus.112075

**Published:** 2026-07-05

**Authors:** Thaleia Angelopoulou, Georgios Manomenidis, Thalia Bellali

**Affiliations:** 1 Medical School, National and Kapodistrian University of Athens, Athens, GRC; 2 Nursing, Democritus University of Thrace, Alexandroupolis, GRC; 3 Health Sciences, European University Cyprus, Nicosia, CYP

**Keywords:** audiovisual distraction technique, behavior management techniques, child anxiety, child behavior, child cooperation, dental care, dental fear and anxiety, dental treatment related anxiety, pediatric and preventive dentistry, tell-show-do technique

## Abstract

Background: Dental anxiety in children is a multifactorial condition influenced by biological, psychological, and environmental determinants. It is commonly associated with uncooperative behavior, compromised treatment outcomes, and negative attitudes toward dental care. To address these challenges, a wide range of non-pharmacological behavior management techniques have been developed and applied in pediatric dentistry, including Tell-Show-Do (TSD) and Audiovisual Distraction (AVD), both of which aim to reduce dental anxiety and enhance cooperative behavior during treatment. Despite their extensive clinical use, comparative evidence on their effectiveness in Greek pediatric patients remains limited.

Methods: A prospective, non-randomized, comparative exploratory study was conducted in a private dental clinic in Athens, Greece, involving 50 children aged 4-15 years, who were allocated to the TSD or AVD group using a non-randomized allocation procedure intended to balance group sizes; allocation concealment was not implemented. Children’s anxiety and cooperative behavior during dental treatment were assessed in real time by an observer not involved in the clinical procedure, using the Venham Behavior Rating Scale (VBRS) and the Venham Clinical Anxiety Scale (VCAS). Demographic and procedural variables were also collected. Statistical analysis was performed using descriptive statistics and the Mann-Whitney U test, with significance set at p < 0.05. Between-group differences were reported with effect sizes (Cliff’s delta) and 95% confidence intervals to quantify magnitude and precision.

Results: Fifty children (mean age 9.8 ± 2.5 years) participated in this study. Both techniques were associated with low anxiety levels and improved cooperation during dental treatment. No statistically significant associations were observed in this sample; however, the study may be underpowered to detect small-to-moderate effects, estimates were imprecise, and residual confounding could not be excluded.

Conclusions: No statistically significant between-group differences were detected in this small, non-randomized clinic sample; equivalence cannot be inferred. These findings contribute population-specific data from Greek children that may support individualized behavior management in routine clinical settings and guide future, larger comparative studies.

## Introduction

Dental anxiety, which often leads to behavior and cooperation difficulties, remains a well-recognized clinical challenge in the field of pediatric dentistry. In children, anxiety typically presents as avoidance of dental care, disruptive or uncooperative behavior, and excessive fear responses toward dental procedures [1]. These reactions not only complicate the delivery of dental care and compromise treatment outcomes but also contribute to the development of unfavorable attitudes toward oral health, potentially resulting in avoidance of future dental care and long-term negative consequences for both oral and general health in young patients [2]. Evidence in the literature indicates that early dental visits and regular exposure to non-invasive preventive procedures in a dental setting can familiarize children with the clinical environment and reduce the likelihood of developing dental fear and anxiety [3].

Dental anxiety arises from the interaction of multiple factors. These include demographic variables such as age and sex [4]; individual characteristics such as temperament [5] and genetic predisposition [6]; and prior negative experiences [7] and the type or invasiveness of the dental procedure performed [8]. In addition, parental anxiety [9] and cultural background [10] may also significantly influence and shape how children perceive and respond to oral health care from a very young age. Investigation of these intrinsic and contextual determinants provides valuable insights for developing targeted psychological and clinical intervention strategies, with the impact of these strategies on children’s anxiety levels and behavioral responses evaluated using a wide range of assessment tools [11].

Given the fact that epidemiological studies report a wide range of prevalence estimates of dental anxiety in children and adolescents, varying from approximately 8% to 12% to over 40% [12,13] depending on methodological approaches, sample characteristics, and cultural context, the need for the systematic implementation of evidence-based behavior management techniques that can effectively reduce dental anxiety, enhance cooperation, and optimize treatment outcomes, while also encouraging early preventive attendance and positive conditioning within the dental setting, is highlighted as a well-established priority in contemporary pediatric dentistry.

Dental treatment in children may require pharmacological sedation, particularly in cases where anxiety, fear, and limited cooperation compromise the feasibility or safety of dental procedures [14]; however, over the years, pediatric dentistry has primarily focused on the development of non-pharmacological behavior management techniques, aiming to promote favorable conditioning toward dental care. Hence, multiple behavior management techniques have been described and investigated in pediatric dentistry, including Tell-Show-Do (TSD), positive reinforcement, modeling, voice control, and audiovisual distraction (AVD) [15]. Among these, TSD and AVD are consistently reported as the most frequently applied techniques, with both demonstrating satisfactory effectiveness in reducing dental anxiety and promoting cooperative behavior [16,17].

In the TSD technique, dentists provide children with a simplified, age-appropriate explanation of the dental procedure, followed by a child-friendly demonstration of the planned intervention [18], whereas the AVD technique employs audiovisual stimuli to divert the child’s attention away from dental stimuli during treatment [19]. Both approaches are widely regarded as ethically acceptable and well tolerated in pediatric dental practice. Through gradual familiarization with dental procedures, TSD fosters familiarity and predictability, while AVD offers a simple, non-invasive distraction that can improve cooperation and alleviate anxiety during treatment [16-19].

Recent systematic reviews and meta-analyses have critically evaluated non-pharmacological behavior management techniques in pediatric dentistry, confirming their overall effectiveness in reducing dental anxiety and improving cooperation in children [15]. However, these analyses also emphasize important limitations in the current evidence base, including substantial heterogeneity in study designs, intervention protocols, outcome measures, and clinical settings [20]. Most available studies evaluate individual techniques in isolation or in non-comparative designs, whereas direct head-to-head comparative evidence between widely used approaches, such as TSD and AVD, remains limited. These gaps highlight the need for comparative studies conducted under routine clinical conditions in order to provide more clinically relevant evidence regarding the relative effectiveness of commonly applied behavior management techniques. Despite their widespread use in pediatric dentistry, comparative clinical data on the application of TSD and AVD within the Greek pediatric population also remain limited.

In Greece, parental perceptions and expectations have been shown to significantly influence children’s behavior in the dental setting, highlighting the importance of behavior management approaches that are both culturally appropriate and acceptable to parents [21,22]. Beyond geographical context, few studies have examined the performance of these behavior management techniques when implemented within routine private dental practice, where variations in procedure type and clinical workflow may influence children’s anxiety and behavioral responses. To address this gap, the present prospective, non-randomized comparative study was designed to compare the effectiveness of TSD and AVD in managing children’s anxiety and behavior during dental treatment in a Greek clinical setting and to provide data relevant to everyday clinical practice. Based on prior research indicating promising outcomes for distraction-based techniques [16-19], it was hypothesized that AVD would be associated with lower anxiety and improved cooperative behavior compared with TSD during dental treatment.

The primary objective of this prospective, non-randomized comparative study was to compare the effectiveness of TSD and AVD in terms of two co-primary outcomes: children’s cooperative behavior during dental treatment, assessed using the Venham Behavior Rating Scale (VBRS), and children’s clinical dental anxiety, assessed using the Venham Clinical Anxiety Scale (VCAS). Age, sex, and type of dental procedure were included as covariates and examined in exploratory analyses to assess their potential influence on these outcomes. Based on prior evidence suggesting favorable effects of distraction-based techniques, we hypothesized that children receiving AVD would demonstrate lower VCAS scores and more favorable VBRS scores than children receiving TSD during dental treatment.

## Materials and methods

Study design and source of data

A prospective non-randomized comparative study was conducted from April to May 2025 in a private dental clinic in Athens, Greece. The study’s protocol was reviewed and approved by the Ethics Committee of the European University of Cyprus (approval number: EUC ETHICS COMMITTEE. 2025-51; approval date: April 4, 2025). The study is reported in accordance with the TREND [23] reporting guideline for non-randomized evaluations.

Sample size and characteristics

The sample size was estimated using G*Power, Version 3.1.9.2 (Heinrich Heine University Düsseldorf, Düsseldorf, Germany), based on data from previous studies. An a priori power analysis for t-tests of independent means indicated a required total sample size of 40 participants (20 per group), with parameters set at an α error probability of 0.05 and a β error probability of 0.02. Given the variability in reported effect sizes across studies and the lack of adequate data in the target population, the present study was not powered to detect small-to-moderate differences and should be considered exploratory. To account for potential dropouts and exclusions, a larger sample was initially screened. During the data collection period, 72 children attending the clinic for routine dental treatment were consecutively invited to participate and assessed for eligibility; of these, 50 met the inclusion criteria and were ultimately enrolled in the study. Inclusion criteria were children aged 4-15 years who were physically and mentally healthy. Exclusion criteria included children with systemic, developmental, or psychiatric disorders, children receiving pharmacological treatment that could influence behavior, and emergency cases requiring urgent dental intervention.

Procedure

Each child’s guardian was informed about the study and was provided with an overview and a clear explanation of the study objectives and procedures. Guardians were informed about their right to refuse participation or withdraw consent at any time without any consequences. Written informed consent was obtained from all guardians before enrollment, and participating children were assigned to one of two study groups. Participants were assigned to TSD or AVD using a non-randomized allocation procedure based on the order of clinical appointments, with a draw-based assignment to balance group sizes (1:1). The non-randomized allocation approach was selected due to pragmatic constraints inherent to a private clinical setting, where strict randomization could disrupt appointment scheduling, clinical workflow, and treatment delivery. Therefore, a structured draw-based allocation procedure was applied to minimize between-group imbalance while preserving feasibility within routine practice. The draw was performed by the investigator (T.A.) after eligibility was confirmed. Allocation concealment was not used, and the treating clinician was aware of group assignment at the time of intervention delivery.

In Group 1, the dentist applied the TSD technique to improve cooperation and reduce anxiety (TSD group). In Group 2, the dentist used the AVD method, projecting animated cartoons on a ceiling-mounted television positioned above the dental chair throughout the procedure (AVD group). The animated cartoons were selected by the child from a predefined set of age-appropriate options, allowing limited variability while maintaining overall standardization. All interventions were delivered by the same pediatric dentist with standardized clinical experience to ensure consistency across participants. Dental procedures included scaling and polishing, followed by topical fluoride application, composite resin restorations, tooth extractions, and fissure sealant placement. Both behavior management techniques evaluated in this study were non-invasive and widely used in pediatric dental practice; therefore, no adverse events were anticipated during their application.

TSD protocol applied in Group 1

Clinical operator: A single pediatric dentist performed all procedures (same operator for all participants).

Application timing: TSD was implemented immediately before and during the dental procedures (scaling and polishing, followed by topical fluoride application, composite resin restorations, tooth extractions, and fissure sealant placement).

Technique description: The technique followed a structured three-step protocol, including an age-appropriate verbal explanation of the upcoming procedure using standardized child-friendly terms (e.g. “cleaning tooth” and “water spray”) (Tell); demonstration of dental instruments (mirror, suction, and handpiece) and procedural steps with visual presentation outside the oral cavity (Show); followed by execution of the procedure as previously explained and demonstrated (Do).

Aim: Familiarization with the procedure and reduction of anticipatory anxiety and unpredictability-related fear.

AVD protocol applied in Group 2

Clinical operator: A single pediatric dentist performed all procedures (same operator for all participants).

Application timing: AVD was implemented immediately before and during the dental procedures (scaling and polishing, followed by topical fluoride application, composite resin restorations, tooth extractions, and fissure sealant placement).

Technique description: The technique involved continuous AVD using animated cartoons projected on a ceiling-mounted television positioned above the dental chair, with content selected by the child from a predefined set of age-appropriate options.

Aim: Redirection of the child’s attention away from the clinical procedure, resulting in reduced awareness of the dental setting and, thus, decreased anxiety responses.

Data collection

During treatment, a researcher (T.A.) not involved in the clinical procedures served as an observer and collected data using standardized printed recording forms. These forms included demographic variables (age and sex), type of dental procedure performed, and the two outcome measures (VBRS) [24] and (VCAS) [24]. Each printed form was coded with a unique participant identifier to ensure anonymity. The observer closely observed, monitored, and recorded each child’s behavioral and anxiety responses throughout the treatment session. VBRS and VCAS ratings were completed in real time, enabling objective quantification of outcomes relative to the applied technique. The observer applied the standardized scoring criteria of the VBRS and VCAS; although no formal calibration or intra-observer reliability assessment was conducted, both scales have consistently demonstrated high inter-rater reliability and reproducibility. Participants in both groups were treated in a single clinical visit following application of the assigned technique. Given the nature of the interventions, blinding the observer to group allocation was not feasible.

**Figure 1:**
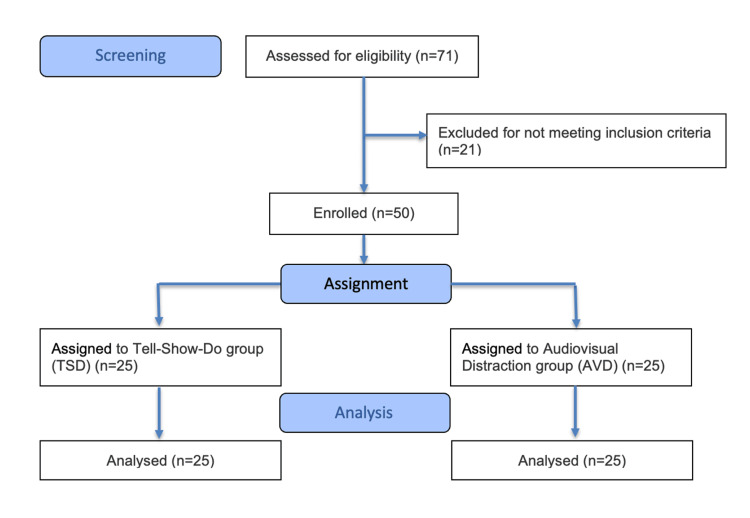
Participant flow diagram. Note: Participant flow diagram prepared by the authors; structure informed by TREND reporting guidance [23].

Following data collection, printed forms were scanned, digitized, and securely stored in a restricted-access storage area, accessible only to the researcher and the supervising professor. Both physical and digital data were scheduled for permanent destruction upon completion of the study. Figure 1 presents the participant flow diagram illustrating the study's recruitment, allocation, and analysis process.

Outcome measures

The primary outcomes of this study were reductions in dental anxiety and improvements in cooperation during dental treatment, as assessed using the VBRS and the VCAS. The Venham scales (VBRS and VCAS) have demonstrated good inter-rater reliability and consistent performance in pediatric dental settings, with studies reporting high agreement between independent observers and stable results across repeated assessments [24]. The secondary outcomes included associations with specific factors: participants’ age and sex as well as the type of dental procedure performed.

To evaluate children's level of cooperation during treatment, VBRS was employed, and the two tested behavior management techniques (TSD and AVD) were implemented. This tool comprises six ordinal categories, with responses ranging from 0 (total cooperation, best possible working conditions, and no crying or physical protest) to 5 (general protest, no compliance or cooperation, and physical restraint required), reflecting a progressive loss of cooperation. Higher scores indicate poorer cooperation and reduced effectiveness of the applied behavior management technique. To assess children’s anxiety levels during dental treatment, VCAS was used while TSD and AVD were applied. VCAS consists of six categories from 0 (relaxed: smiling, willing to cooperate, able to converse, and displays behavior desired by the dentist) to 5 (out of contact: hard, loud swearing, screaming, unable to listen, trying to escape, and physical restraint required), reflecting progressively higher anxiety levels. Similarly to the VBRS, higher scores denote greater anxiety and lower effectiveness of the applied anxiety management technique.

Statistical analysis

Data were entered into IBM SPSS Statistics for Windows, Version 24 (Released 2016; IBM Corp., Armonk, New York). Categorical variables were summarized as frequencies and percentages. The ordinal outcomes (VBRS and VCAS) were summarized as medians with interquartile ranges and score distributions. Continuous variables, where applicable, were summarized as means with standard deviations. Baseline imbalance between groups was assessed using standardized mean differences (SMDs). Between-group comparisons for VBRS and VCAS were performed using the Mann-Whitney U test and were quantified using Cliff’s delta with bootstrap 95% confidence intervals (2000-5000 resamples). Associations of outcomes with age, sex, and procedure type were considered exploratory. As an exploratory sensitivity analysis, proportional-odds ordinal logistic regression models adjusting for intervention group, age, sex, and procedure type were fitted. No multiplicity adjustment was applied because secondary and subgroup analyses were exploratory. All tests were two-sided, and statistical significance was set at p < 0.05.

## Results

Participant recruitment and data collection were conducted between April and May 2025, with all outcomes assessed during a single clinical visit. Using the approach described above, 50 eligible children were enrolled and assigned to the two study groups: 25 to the TSD group and 25 to the AVD group. All participants received the allocated intervention and were included in the analysis of the primary and secondary outcomes.

Baseline demographic and clinical characteristics by intervention group are presented in Table 1. The median age was 9.0 years (9.0-11.5) in the TSD group and 10.0 years (7.0-11.5) in the AVD group (absolute SMD = 0.16). Females accounted for 64.0% of the TSD group and 52.0% of the AVD group (absolute SMD = 0.24). The most frequent dental procedure in both groups was scaling and polishing with fluoride application, while the distribution of the remaining procedures was similar overall. Absolute SMDs were small for procedure type (0.08-0.12), suggesting limited baseline imbalance.

**Table 1:** Baseline demographic and clinical characteristics by intervention group. Data are presented as median (Q1-Q3) or n (%) as appropriate. Absolute standardized mean differences (SMD) are shown, with larger values indicating greater between-group imbalance.

Variable	TSD (n = 25)	AVD (n = 25)	Absolute SMD
Age (years)	9.0 (9.0-11.5)	10.0 (7.0-11.5)	0.16
Sex			
Female, n (%)	16 (64.0)	13 (52.0)	0.24
Male, n (%)	9 (36.0)	12 (48.0)	-
Type of dental procedure			
Scaling and polishing/fluoride application, n (%)	13 (52.0)	12 (48.0)	0.08
Composite resin restoration, n (%)	3 (12.0)	4 (16.0)	0.12
Tooth extraction, n (%)	5 (20.0)	4 (16.0)	0.10
Pit and fissure sealants, n (%)	4 (16.0)	5 (20.0)	0.10

Between-group comparisons of the two co-primary ordinal outcomes are presented in Table 2. For the VBRS, the median score was 1.0 (Q1-Q3: 1.0-3.0) in the TSD group and 1.0 (Q1-Q3: 0.0-4.0) in the AVD group. The between-group difference was not statistically significant (Mann-Whitney U = 327.0, p = 0.780), and the corresponding effect size was negligible (Cliff’s delta = 0.05, 95% CI: -0.29 to 0.37). For the VCAS, the median score was 1.0 (Q1-Q3: 1.0-2.0) in the TSD group and 1.0 (Q1-Q3: 0.0-4.0) in the AVD group. Again, no statistically significant between-group difference was detected (Mann-Whitney U = 318.5, p = 0.913), with a negligible effect size (Cliff’s delta = 0.02, 95% CI: -0.31-0.35). Overall, these findings indicate no evidence of a meaningful difference between the two techniques in observed cooperation or clinical anxiety in this sample; however, the study may be underpowered to detect small-to-moderate differences between groups. These score ranges correspond predominantly to mild behavioral responses and low-to-moderate levels of clinical anxiety, suggesting that most children were able to cooperate with treatment with minimal disruption to chairside workflow.

**Table 2:** Between-group comparison of the co-primary ordinal outcomes (VBRS and VCAS). VBRS: Venham Behavior Rating Scale; VCAS: Venham Clinical Anxiety Scale.

Outcome	TSD median (Q1-Q3)	AVD (n = 25) median (Q1-Q3)	Mann-Whitney U	Cliff’s delta	95% CI	p-value
VBRS	1.0 (1.0-3.0)	1.0 (0.0-4.0)	327.0	0.05	-0.29-0.37	0.780
VCAS	1.0 (1.0-2.0)	1.0 (0.0-4.0)	318.5	0.02	-0.31-0.35	0.913

The score distributions for VBRS and VCAS by intervention group are presented in Table 3. In the TSD group, VBRS scores were most frequently at 1 (32.0%), followed by 0 and 2 (20.0% each), whereas in the AVD group, the most common VBRS score was 0 (48.0%). For VCAS, score 1 was the most frequent category in the TSD group (36.0%), while score 0 was the most frequent category in the AVD group (40.0%). In both outcomes, higher scores were observed in a minority of children, although some heterogeneity in score distributions was evident across the two intervention groups. Overall, the observed distribution of scores indicates that, in most cases, the applied behavior management techniques were sufficient to maintain acceptable working conditions, with only a minority of children exhibiting behaviors that could significantly interfere with treatment delivery.

**Table 3:** Distribution of VBRS and VCAS scores by intervention group. VBRS: Venham Behavior Rating Scale; VCAS: Venham Clinical Anxiety Scale.

Score	TSD (n = 25), n (%)	AVD (n = 25), n (%)
VBRS		
0	5 (20.0)	12 (48.0)
1	8 (32.0)	1 (4.0)
2	5 (20.0)	3 (12.0)
3	4 (16.0)	1 (4.0)
4	3 (12.0)	5 (20.0)
5	0 (0.0)	3 (12.0)
VCAS		
0	4 (16.0)	10 (40.0)
1	9 (36.0)	3 (12.0)
2	6 (24.0)	3 (12.0)
3	3 (12.0)	1 (4.0)
4	3 (12.0)	5 (20.0)
5	0 (0.0)	3 (12.0)

Exploratory proportional-odds ordinal logistic regression models adjusting for intervention group, age, sex, and type of dental procedure are presented in Table 4. After adjustment, allocation to AVD versus TSD was not significantly associated with either VBRS (aOR = 1.06, 95% CI: 0.38-2.93, p = 0.916) or VCAS (aOR = 1.07, 95% CI: 0.39-2.96, p = 0.890). Age and sex were also not significantly associated with either outcome. Compared with scaling and polishing with fluoride application, composite resin restoration was associated with higher odds of less favorable scores on both VBRS (aOR = 6.87, 95% CI: 1.30-36.38, p = 0.024) and VCAS (aOR = 6.48, 95% CI: 1.23-34.12, p = 0.027). No statistically significant associations were observed with tooth extraction or pit-and-fissure sealants.

**Table 4:** Exploratory proportional-odds ordinal logistic regression models for VBRS and VCAS. Adjusted proportional-odds ordinal logistic regression models were fitted separately for VBRS and VCAS. Results are presented as adjusted odds ratios (aOR) with 95% confidence intervals. Reference categories were TSD, female sex, and scaling and polishing with fluoride application. Higher scores indicate poorer cooperation and greater clinical anxiety. TSD: Tell-Show-Do; VBRS: Venham Behavior Rating Scale; VCAS: Venham Clinical Anxiety Scale.

Predictor	Adjusted OR (95% CI) for VBRS	p-value	Adjusted OR (95% CI) for VCAS	p-value
AVD vs TSD	1.06 (0.38-2.93)	0.916	1.07 (0.39-2.96)	0.890
Age (per year)	0.95 (0.76-1.19)	0.668	0.89 (0.71-1.11)	0.296
Male vs female	0.69 (0.24-1.98)	0.490	0.68 (0.24-1.94)	0.471
Composite restoration vs scaling/fluoride	6.87 (1.30-36.38)	0.024	6.48 (1.23-34.12)	0.027
Tooth extraction vs scaling/fluoride	1.49 (0.37-5.98)	0.571	1.68 (0.42-6.75)	0.465
Pit and fissure sealants vs scaling/fluoride	0.45 (0.11-1.92)	0.282	0.67 (0.17-2.71)	0.574

## Discussion

The present study compared the effectiveness of two widely used non-pharmacological behavior management techniques: TSD and AVD. The primary objective of this study was to assess dental anxiety and patient cooperation during dental treatment, measured using the VBRS and the VCAS. In addition, the potential influence of demographic and clinical variables, including age, sex, and type of dental procedure, was examined using adjusted analytical models.

The findings of this study indicated that both techniques were associated with relatively low anxiety and acceptable cooperation. No statistically significant differences were observed between the two techniques for either VBRS or VCAS, and effect size estimates (Cliff’s delta) were negligible, further supporting the absence of a clinically meaningful difference between the interventions. These results persisted after adjustment for age, sex, and type of dental procedure using proportional-odds ordinal logistic regression models, suggesting that the lack of between-group differences was not attributable to measured confounding by these variables. These findings are consistent with several previous clinical studies reporting comparable outcomes for TSD and AVD [25,26]. Conversely, most studies in the existing literature have documented greater effectiveness of AVD when compared with other methods [16,17].

The absence of statistically significant differences between the two techniques may have been influenced by residual or unmeasured confounding and heterogeneity within the sample, despite adjustment for key variables. Variables such as age and type of dental procedure may have affected the observed outcomes and potentially masked true between-group differences. To further explore whether the absence of between-group differences might be influenced by underlying clinical or demographic factors, adjusted analyses were conducted. Notably, these analyses identified the type of dental procedure as a significant determinant of both behavioral response and clinical anxiety. Specifically, composite resin restorations were associated with significantly higher odds of less favorable VBRS and VCAS scores compared with scaling and polishing with fluoride application. In contrast, no statistically significant associations were observed for tooth extraction or pit and fissure sealants. These findings suggest that the invasiveness of the procedure and treatment-related stimuli may have a stronger influence on children’s behavioral and emotional responses than the behavior management technique itself. These observations are consistent with prior studies indicating greater stress responses to invasive treatments, often attributed to longer treatment durations or due to drilling sounds and the use of injections [8].

Furthermore, it is well established that dental anxiety results from the dynamic interaction of many biological, psychological, and environmental determinants [5-7,9,10]. Demographic factors, including age and sex [4], and the type and invasiveness of dental procedures performed [8], have been associated with unfavorable responses to dental treatment, as reflected in heightened anxiety and reduced cooperation. Although in the present study, female participants tended to display higher levels of anxiety and lower levels of cooperation compared to the male participants, a finding consistent with previous studies [27], this association did not reach statistical significance after adjustment. These results are consistent with evidence from other studies that have also reported no significant difference between the two genders [28,29,30]. In terms of age, even though younger children (4-9 years old) participating in this trial tended to present with higher anxiety levels and more disruptive behaviors compared to the older age groups, these results also did not reach statistical significance after adjustment. This finding should be interpreted in the context of the relatively broad developmental age range of the sample, since older children may have greater capacity to understand procedural explanations, regulate anxiety, and cooperate during dental treatment. However, because age was not significantly associated with VBRS or VCAS scores in the adjusted analyses, the observed behavioral and anxiety patterns cannot be attributed specifically to the younger subset of participants. Future studies with narrower age groups or age-stratified analyses are needed to clarify whether the relative effectiveness of TSD and AVD differs across developmental stages. Some studies in the literature suggest that developmental factors related to cognitive and emotional regulation capacity enable older children to process instructions and the potential benefits of applying behavior management techniques [12,13]. In contrast, other studies report no association between age and anxiety outcomes [4]. Heterogeneity in findings could reflect differences in study design and sample characteristics, such as individual psychological factors, baseline anxiety levels, temperament, or previous dental trauma.

Both behavior management techniques were implemented consistently within the practice's routine clinical workflow. The TSD technique was delivered in a structured, stepwise manner, while AVD was applied using continuous audiovisual stimuli throughout the procedure. Although no major deviations from the intended protocols were observed, minor variations in individual patient behavior, attention span, and procedure duration occasionally occurred and may have influenced intervention delivery; however, these variations were minimal and did not appear to affect the overall consistency of application or the observed outcomes.

Overall, the results of this study further highlight the complex nature of dental anxiety. Beyond age and gender, broader sociocultural influences and parental attitudes also appear to play a critical role in shaping children’s responses during dental treatment [9,29]. In this context, the availability of population-specific data becomes particularly important. Although the influence of parental perceptions on children’s behavior in dental settings in Greece has been investigated [21,22], comparative clinical data regarding the application of TSD and AVD in the Greek pediatric population remain limited. Although pharmacological approaches such as inhalation sedation are well established internationally, their integration into routine pediatric dental practice in Greece remains comparatively limited due to restrictive regulatory frameworks that were in effect until recently. This fact, therefore, further emphasizes the importance of non-pharmacological behavior management strategies in Greek settings. The present study therefore adds context-specific evidence from a Greek pediatric population treated in a routine private clinical setting, an area that has not been adequately investigated in the existing literature, and thereby provides a novel contribution with clinically relevant insight into the use of TSD and AVD under everyday practice conditions. From a clinical perspective, both TSD and AVD appear to be effective behavior management options in routine pediatric dental practice, particularly when applied flexibly according to the child’s age, anxiety level, procedure type, and chairside response. For less invasive procedures, either technique may be sufficient to maintain cooperation and reduce anxiety, whereas more invasive or longer procedures may require closer behavioral monitoring and additional preparatory communication. These findings support an individualized, chairside-adaptive approach to behavior management.

Several limitations should be considered when interpreting the results of the present prospective non-randomized comparative study, and the absence of statistically significant differences between the two techniques should be interpreted with caution. Possible explanations include methodological limitations, such as non-randomized allocation and heterogeneity in key variables (e.g., age and type of dental procedure), which may have introduced selection bias and residual confounding despite efforts to balance group sizes. The absence of randomization and allocation concealment, in particular, represents a major inherent limitation of this study design that may have introduced selection bias. This approach was adopted due to pragmatic constraints related to the clinical setting; however, it limits internal validity and should be considered when interpreting the findings. These findings should also be interpreted within the context of a single private dental practice in Greece, which may differ substantially from public healthcare settings in terms of patient population characteristics, access to care, organizational structure, and clinical workflow.

In private practice settings, patients may present with different socioeconomic backgrounds, levels of prior dental exposure, and patterns of healthcare utilization compared with those attending public clinics, where access, waiting times, and treatment priorities may vary. Moreover, differences in healthcare systems and cultural contexts may influence children’s behavioral responses, parental expectations, and acceptance of behavior management techniques, thereby limiting the extent to which the present findings can be generalized to other clinical environments and populations. The conduct of the study in a single private dental clinic may limit the external validity and generalizability of the findings, as patient characteristics, clinical practices, and healthcare delivery conditions may vary across different clinical settings and populations. Additionally, the clinical interventions were not restricted to a single type of dental procedure; heterogeneity in procedure invasiveness (e.g., prophylaxis versus restorations or extractions) may have influenced anxiety and behavior outcomes and potentially obscured between-group differences. Importantly, the included procedures were not equivalent in terms of type, invasiveness, and clinical complexity, ranging from preventive interventions such as fluoride application to more invasive procedures such as composite restorations and extractions, nor were they evenly distributed across participants, which may have introduced additional variability in behavioral and anxiety responses. This heterogeneity reflects the variability inherent in routine clinical practice. However, it also represents a substantial source of clinical heterogeneity and a potential confounding factor that constitutes an important limitation of the study.

The relatively small sample size from a single private practice setting, intra-group variability and heterogeneity (e.g., age variations), and a lack of assessment of individual psychological factors, such as baseline anxiety levels, temperament, or previous dental trauma, as well as other important potential confounding variables, including previous dental experiences, parental anxiety and influence, and socioeconomic or educational background, further limit the precision and generalizability of the findings and may have resulted in residual confounding and increased the risk of bias in the observed associations. Not accounting for these variables constitutes a major limitation of this study, given their established importance in pediatric behavioral research. In addition, the limited sample size suggests that the study may be underpowered to detect small-to-moderate effects, thereby restricting the interpretability of the findings and limiting the ability to draw definitive conclusions regarding the comparative effectiveness of the interventions. Moreover, outcomes were measured using observer-rated ordinal scales (VBRS and VCAS), and blinding of the assessor was not feasible in this setting, which may have increased the risk of outcome assessment (measurement) bias, and although standardized and well-validated instruments were used, with previous studies reporting high inter-rater reliability and reproducibility for the Venham scales, the absence of formal calibration or intra-observer reliability assessment in the present study may have introduced measurement variability. Notably, a subset of children appeared unresponsive to either behavior management approach, highlighting the overall limitations of standardized techniques widely used in pediatric dentistry.

Furthermore, parental perspectives, including parents’ perceptions of the intervention and their potential influence on children’s behavioral responses, which could provide a more holistic understanding of children’s reactions during dental treatment, were not considered, and the absence of follow-up assessments limits the evaluation of the sustained effectiveness of these techniques over time. Future research should, therefore, focus on larger, multicenter studies conducted in academic settings, incorporate qualitative data such as parental interviews and questionnaires, consider stratification or adjustment for procedure type and other key confounders, and explore emerging audiovisual technologies, including virtual reality or mobile application-based interventions. Strengthening the evidence base in this direction is essential, as it supports the development of more individualized behavior management strategies that can improve treatment outcomes, promote positive dental experiences during childhood, and foster long-term trust in oral health care.

## Conclusions

In conclusion, the present prospective comparative study found that both TSD and AVD techniques were associated with generally low observed anxiety and acceptable levels of cooperation, with no statistically significant between-group differences detected. Both techniques appear to represent acceptable behavior management approaches whose effectiveness may be context-dependent in routine clinical practice.

Several limitations of this study constrain the interpretability and generalizability of its findings. Equivalence between the two techniques cannot be concluded; further research with larger sample sizes, multicenter settings, and appropriate adjustment for potential confounders is needed to establish the comparative effectiveness of these behavior management techniques.
